# Carcass Type Affects Local Scavenger Guilds More than Habitat Connectivity

**DOI:** 10.1371/journal.pone.0147798

**Published:** 2016-02-17

**Authors:** Zachary H. Olson, James C. Beasley, Olin E. Rhodes

**Affiliations:** 1 Department of Psychology, University of New England, 11 Hills Beach Road, Biddeford, Maine, United States of America; 2 Savannah River Ecology Laboratory, University of Georgia, Aiken, South Carolina, United States of America; 3 D. B. Warnell School of Forestry and Natural Resources, University of Georgia, Athens, Georgia, United States of America; 4 Odum School of Ecology, University of Georgia, Athens, Georgia, United States of America; University of Lleida, SPAIN

## Abstract

Scavengers and decomposers provide an important ecosystem service by removing carrion from the environment. Scavenging and decomposition are known to be temperature-dependent, but less is known about other factors that might affect carrion removal. We conducted an experiment in which we manipulated combinations of patch connectivity and carcass type, and measured responses by local scavenger guilds along with aspects of carcass depletion. We conducted twelve, 1-month trials in which five raccoon (*Procyon lotor*), Virginia opossum (*Didelphis virginiana*), and domestic rabbit (*Oryctolagus* spp.) carcasses (180 trials total) were monitored using remote cameras in 21 forest patches in north-central Indiana, USA. Of 143 trials with complete data, we identified fifteen species of vertebrate scavengers divided evenly among mammalian (N = 8) and avian species (N = 7). Fourteen carcasses (9.8%) were completely consumed by invertebrates, vertebrates exhibited scavenging behavior at 125 carcasses (87.4%), and four carcasses (2.8%) remained unexploited. Among vertebrates, mammals scavenged 106 carcasses, birds scavenged 88 carcasses, and mammals and birds scavenged 69 carcasses. Contrary to our expectations, carcass type affected the assemblage of local scavenger guilds more than patch connectivity. However, neither carcass type nor connectivity explained variation in temporal measures of carcass removal. Interestingly, increasing richness of local vertebrate scavenger guilds contributed moderately to rates of carrion removal (≈6% per species increase in richness). We conclude that scavenger-specific differences in carrion utilization exist among carcass types and that reliable delivery of carrion removal as an ecosystem service may depend on robust vertebrate and invertebrate communities acting synergistically.

## Introduction

Scavengers and decomposers provide an important ecosystem service by removing carrion, or dead animal flesh, from the environment. At one end along a spectrum of possible species assemblages that assimilates carrion resources, a carcass can be decomposed entirely by invertebrate and microbial action. When a carcass is removed by invertebrates and microbes, a flush of unincorporated nutrients is released, and these nutrients subsidize local primary production near the site of the carcass [1,2]. At the other end of the spectrum, many carcasses are removed by vertebrate scavengers largely to the exclusion and competitive disadvantage of invertebrate and microbial decomposers [3,4]. When compared to removal by invertebrate and microbial decomposition, carcass removal by scavengers results in greater diffusion of nutrients away from the site of the carcass and maintains carcass energy and nutrients at higher trophic levels within food webs [5]. Ultimately, the trophic fate of the nutrients in a particular carcass is determined by which species assemble into a local guild (e.g., [6]) at that carcass.

Despite potential variation in local guilds at carcasses, carrion removal is accomplished with repeatability across landscapes and through time [3]. The general efficiency of carrion removal is highlighted by the rarity with which animal remains—including human remains—are found intact during forensic and archaeological investigations [7]. However, research has revealed that carrion removal is sensitive to a few key input parameters such as carcass size [8], temperature of the environment [5,9], and perturbations to the vertebrate scavenger community [10].

Carcass size contributes to carrion removal in that larger carrion facilitates the assemblage of a greater richness of scavengers that seem to remove carcasses more quickly [8]. Scavenging and decomposition are also intrinsically tied to temperature dependent biochemical processes [1,11], with temporal aspects of carrion removal proceeding more slowly in cooler micro-sites and colder seasons [11,12]. Lastly, while changes to carrion availability has been shown to alter scavenger population demography and viability (e.g., due to policy decisions involving livestock carcass disposal in Europe; [13–16]), alterations to the structure of vertebrate scavenging communities also have been shown to decrease the rate of carcass removal on the landscape, whether those alterations were unplanned [17] or experimental [10]. Given that the delivery of carcass removal as an ecosystem service is sensitive to changes in vertebrate communities, habitat fragmentation might also be expected to affect how carrion removal is delivered.

Habitat fragmentation has pervasive effects on the structure of animal communities [18]. In fragmented landscapes, animals alter their movement behavior relative to remaining habitats [19,20], which when combined with metapopulation dynamics [21] leads to differences in habitat patch occupancy among species [22]. The primary mechanism driving community assemblage among fragmented habitat patches seems to be physical attributes of the patches, such as habitat patch size and isolation, with physical attributes of the patches being complemented by species ecology (i.e., body size, niche breadth, etc.) in determining community composition in each patch [22]. Patch size and patch isolation are likely to affect carrion removal if they alter the composition of communities from which scavengers and decomposers emerge as local guilds [23]. For example, we might expect more avian scavenging or invertebrate decomposition to occur in smaller, more-isolated habitat patches that contain fewer resident vertebrates that compete for carrion. Indeed, levels of habitat fragmentation have already been linked to the probability that a carcass is removed via decomposition by invertebrates or through scavenging by vertebrates [24–27].

Beyond carcass size, carcass type also likely plays a role in the probability of carcass removal by vertebrate species. For example, vertebrate scavengers have been known to avoid diseased carcasses or those of conspecifics [3,28]. This suggests that carcass size alone does not dictate all scavenging outcomes. Identifying the factors affecting carrion removal is important from the perspective of understanding nutrient cycling, because scavenging and decomposition have different local outcomes on nutrient pools. However, factors affecting the fate of carcasses are also important to identify because carcasses can act as foci for disease transmission on the landscape [29–31]. Parameters associated with temporal aspects of carrion removal such as the amount of time carcass tissues remain available in a given landscape may provide signals of transmission risks for carrion-associated diseases (e.g., [32,33]).

Our objective was to determine the relative importance of patch connectivity and carcass type on two facets of carrion removal: patterns in the species that assemble to feed on carcasses and temporal aspects of carcass removal. To address our objective, we conducted an experiment in which we manipulated combinations of patch connectivity and carcass type, and measured carrion use by local scavenger guilds and temporal aspects of carcass depletion. We tested the following hypotheses: 1) habitat patch connectivity will influence the structure and diversity of local scavenger guilds more than carcass type; 2) carcass type will affect how quickly and to what extent carcasses are removed more than habitat connectivity; and, lastly, 3) changes in local scavenger guilds will drive variation in temporal aspects of carcass removal.

## Methods

### Study area

**Fig 1:**
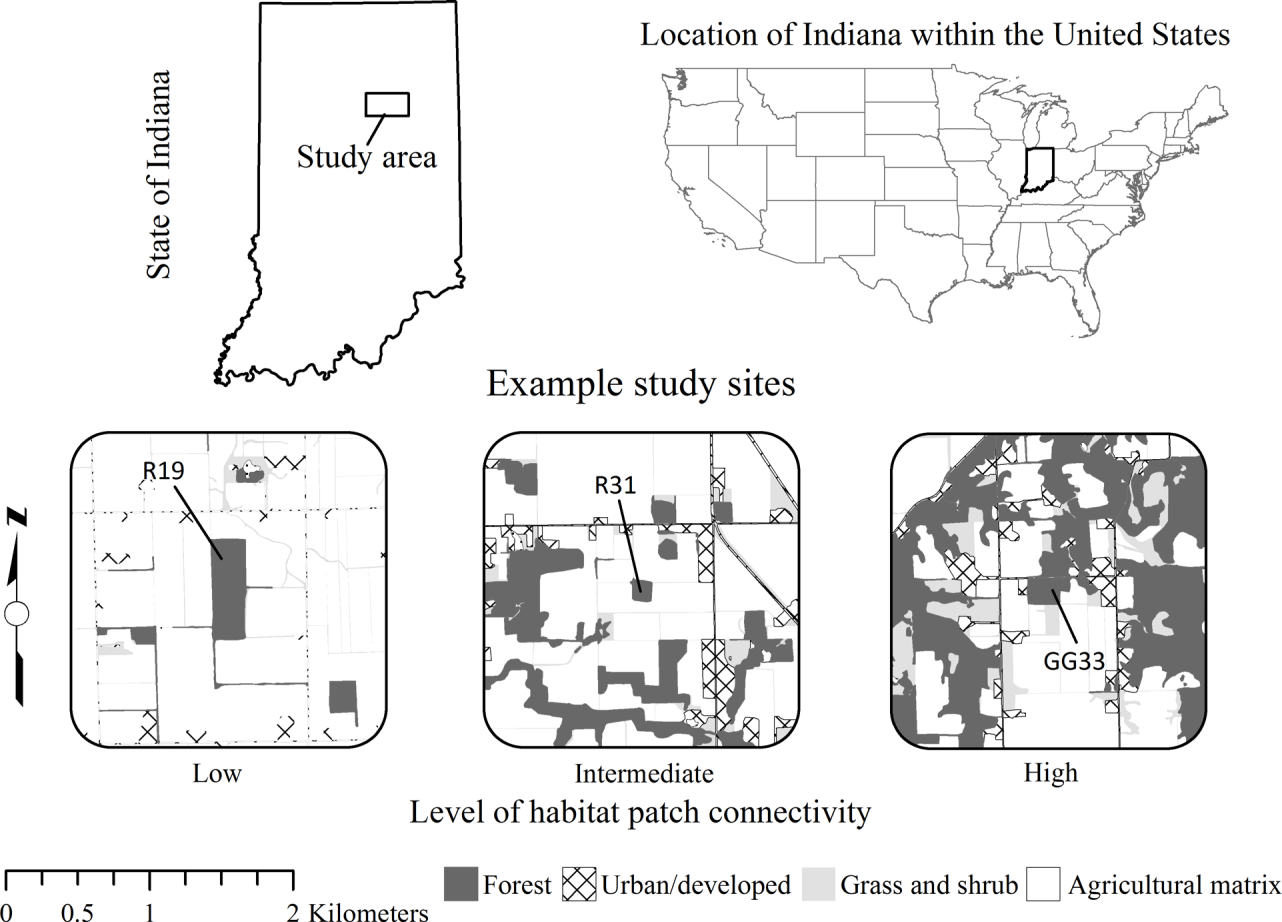
Study area and example study sites. Study sites were small woodlots (i.e., habitat patches) located in a north-central Indiana, USA, study area. Study sites were selected to represent the degrees of patch isolation found on the landscape, and sites were categorized as low, intermediate, and high connectivity. Low connectivity sites averaged 8.3 ± 4.6% (± 1SE) forest habitat in the surrounding 92 ha of land (where 92 ha represents the average home range size of an adult male raccoon in the study area; Beasley et al. 2011), intermediate connectivity sites averaged 13.5 ± 2.5% forest habitat, and high connectivity sites averaged 32.0 ± 2.0% forest habitat in the surrounding 92 ha. Layers used to create this figure are freely available from the USGS National Map Viewer http://viewer.nationalmap.gov/viewer/.

The Upper Wabash River Basin (UWB) in North-Central Indiana, USA, is a region characterized by intense row-crop agricultural activity. Since European settlement, forest cover (primarily oak-hickory-maple [*Quercus-Carya-Acer*]) has been reduced from 87% to only 8% to accommodate the dominant land-use today: production of corn (*Zea mays*) and soybeans (*Glycine max*). Within the UWB our study area spanned 1,165 km^2^ (450 mi^2^; Fig 1), and consisted of 66% agriculture, 15% forest, 6% anthropogenic land, 3% water, 2% shrub land, 1% roads, and 1% fence-row corridors by land-cover [34]. Forested land is heavily fragmented by a matrix of agricultural and other anthropogenic land-uses, creating a sparse network of forest patches that are generally small (<20 ha) and relatively isolated from one another (Fig 1). Large (i.e., >100 ha) forest tracts are rare, but do occur in areas not suitable for cultivation [35]. As such, our study area is representative of many areas where intensive agriculture is the dominant land-use.

Specific study sites included 21 privately-owned forest patches that were distributed throughout the study area and represented the range of patch sizes and degrees of patch isolation present on the landscape [34]. Sites were ordered using an index of proximity (PROX function) generated from 1-m resolution habitat maps using FRAGSTATS v. 3 [36] in a geographic information system. The index is sensitive to patch size and isolation, and assigns higher scores to sites with many large forest patches nearby and lower scores to smaller sites lacking neighbors. We used the index to bin study sites into categories of Low, Intermediate, and High levels of habitat connectivity (Fig 1 and S1 Fig). Although species differ in their responses to habitat fragmentation, previous work in our system demonstrated the importance of patch size and isolation for movement behaviors of the dominant mammalian scavengers ([24]; i.e., raccoon *Procyon lotor* [19] and Virginia opossum *Didelphis virginiana* [20]) and for predicting community membership more broadly [35]. Landowner permission was gained prior to accessing all field sites.

### Field methods

We monitored raccoon, Virginia opossum (hereafter opossum), and rabbit (*Oryctolagus cunnilingus*) carcasses to identify scavengers from September 2008—August 2009. Raccoon and opossum carcasses were chosen because both species are common mesopredators in the study area and were the top and second-most-frequent scavengers, respectively, in previous scavenging trials in this landscape [10]. Domestic rabbit carcasses were intended to represent the native Eastern cottontail rabbit (*Sylvilagus floridanus*), an abundant, medium-sized prey species also common in the study area [37]. Studies using animal carcasses are not subject to Purdue Animal Care and Use Committee (PACUC) review. Thus, no approval was necessary to obtain or use these carcasses in research.

Rabbit carcasses were purchased from a local breeder, and raccoon and opossum carcasses were purchased from a local trapper. The study did not involve endangered or threatened species. All carcasses were whole, had no external damage, and were stored frozen from < 1 day after death until being thawed prior to deployment. We distributed 15 carcasses (five per species) among 21 forest patches during 12 consecutive 1-month trials. The location of each carcass was randomized within a patch. Patches only received one carcass in a given trial, and any remains were removed from patches prior to the start of the next trial. Six patches were dropped from receiving a carcass in each trial in an attempt to minimize scavengers habituating to carcasses (all patches dropped in ≥ 2 trials study wide). To determine the type of carcass that a patch would receive in a given month (if any), we used a pseudo-Latin square design blocked by four seasons and three classes of habitat patch connectivity. Experimental blocks (season x patch connectivity) received 5 replicates of each carcass type for a study-wide total of 180 carcasses (i.e., 60 of each species). Seasons were fall (Sep-Nov), winter (Dec-Feb), spring (Mar-May), and summer (Jun-Aug).

Each carcass was secured loosely to the ground with a small cable snare and metal trapping stake, and monitored for one month using a remote camera (Stealth Cam LLC, Grand Prairie, TX, USA). Cameras were mounted ~1.5 m high on a tree bole approximately 3 m from the carcass. Cameras were programmed to capture a series of three images as quickly as the camera could cycle when triggered by animal movement (5–30 s between images), and then to rest for 30 s before subsequent triggers.

We used the following rules to score images and identify scavengers. Images were scored as “scavenging” events if an animal made contact with the carcass (i.e., appeared to be feeding) and the carcass moved between images. All other images of animals were scored as a “visit.” Images separated in time by > 5 min were scored as independent events, whereas images of the same species separated by ≤ 5 min were scored as adding duration to a scavenging event. We used five minutes as our threshold between independent events, because most consecutive images of the same species occurred within that time (i.e., likely the same individual) and most species switches between consecutive images occurred after five minutes had elapsed.

We documented initial carcass weight, date and time of carcass placement, time to the first vertebrate scavenger (i.e., contact and carcass moved) in hours using image time-stamps and in accumulated degree days ([29]; see below), time to the carcass being opened (i.e., skin perforated) in hours and accumulated degree days, and the scavenger responsible for opening the carcass. To measure carcass depletion we used the complete series of images (often numbering in the thousands per carcass) to estimate visually the percent of edible material remaining at each scavenging event to the nearest 5%, and we documented time to the last recorded scavenger in hours and accumulated degree days. The dry stage of decomposition [1] was considered fully consumed.

Accumulated degree days (ADD) is a measurement commonly used in the study of temperature-dependant processes such as insect development and in forensic applications. We calculated degree days for each day of the study as the difference between the average daily temperature (daily min + max temperature / 2) and a base temperature of 10°C which has entomological relevance [38]. We then tracked the accumulation of degree days for each carcass after it was deployed. For example, a day with an average temperature of 12°C would increase ADD by 2°. Daily minimum and maximum air temperatures were identified for the study area as the average of the values documented by the three nearest weather monitoring stations (5 km south of the study area—USC00129138, in study area—USC00125337, and 10 km north of the study area—USC00126420; www.ncdc.noaa.gov).

### Statistical analyses

#### 1.1 Do connectivity and carcass type affect local scavenger guilds?

We used several measurements in our efforts to quantify patterns of species participation in carcass removal. We considered measures of alpha diversity (i.e., species richness) and beta diversity (i.e., variation in community structure [39]) of local scavenger guilds, complexity of scavenging activity (describes variation in duration of scavenging by species), the identity of the species responsible for opening a carcass, and the number of carcasses monopolized by invertebrates to be components of the pattern of species participation in carcass removal.

To identify local scavenger guild richness, we recorded the number of vertebrate species that scavenged each carcass [6]. To determine if connectivity or carcass type affected richness of local scavenger guilds (i.e., affected alpha diversity), we constructed linear models of richness with connectivity, carcass type, and season as independent variables. We interpreted competitive models (i.e., ΔAIC < 4.0) identified from a suite of models incorporating all possible parameter combinations using a model selection procedure. Model selection throughout was based on minimizing Akaike information criterion (AIC; [40]). All analyses were conducted in R v. 2.15 [41].

To determine if connectivity or carcass type affected beta diversity of local scavenger guilds, we first compiled a site × species matrix in which we noted the presence (1) or absence (0) of scavengers at carcasses (i.e., carcasses were sites and scavengers were species). We constructed pairwise distance matrices (1—Sorensen and 1—Jaccard indices) with function *betadiver*, and modeled connectivity and carcass type against the two distance matrices using permutational multivariate analysis of variance (PERMANOVA) via *adonis* in the vegan package [42]. Sorensen and Jaccard indices were chosen because they exclude joint species absences in the calculation of pair-wise similarity. We permuted dissimilarities within seasons 999 times to assess significance. Because *adonis* models do not accommodate post-hoc analyses, we investigated significant main effects further using principle coordinate analysis (PCO via *cmdscale*). Separately, we modeled dispersion (e.g., multivariate variance) due to connectivity and carcass type around local scavenger guild centroids using function *betadisper* in vegan. Significant F-tests for dispersion were further investigated using post hoc analyses.

We created a second site (carcass) × species (scavenger) matrix using the total duration of scavenging time for each species at each carcass to incorporate temporal aspects of scavenging as a separate assessment of beta diversity among local scavenger guilds. We measured the amount of time each species scavenged each carcass based on the series of pictures recorded during scavenging bouts. Bout times of < 5 minutes were rounded up to 5 minutes (i.e., all bouts lasted at least 5 minutes or 0.083 hours); this minimum aligned with our system of image-scoring. We used the ‘sum-of-LR’ method [43] to model connectivity, carcass type, and season against our measure of the complexity of scavenging activity. The sum-of-LR method makes use of multivariate negative binomial regression to explicitly accommodate the systematic mean-variance relationships common to distance-based indices, and therefore offers a way to avoid confounding any effects due to differences in centroid location with effects due to differences in group dispersions [43]. We visually inspected a plot of model residuals vs. fits to ensure that a negative binomial error distribution was appropriate before we proceeded with model interpretation [43].

We used log-linear models to determine if the frequency with which species were responsible for opening carcasses varied by patch connectivity or carcass type. For this analysis we grouped species into ‘mammal’, ‘bird’, and ‘invertebrate’ categories to eliminate empty cells, and we excluded data from winter trials when invertebrates were inactive. Likelihood ratio tests were used to identify significant effects. Lastly, to determine if the proportion of carcasses monopolized by invertebrates (i.e., opened by invertebrates and no vertebrate scavengers) differed by connectivity or carcass type, we used a series of log-linear models with likelihood ratio tests to identify significant effects.

#### 1.2 Do connectivity and carcass type affect the timing and extent of carrion removal?

We measured four variables that represented the timing and extent of carcass removal: time to first scavenger, time to carcass being opened, time to last scavenger, and the number of carcasses that remained un-depleted. We constructed linear mixed effects models with all subsets of parameter combinations from saturated to the null model to determine if mean time to first scavenger, mean time to carcass being opened, and mean time to last scavenger were affected by connectivity, carcass type, or season using a model selection procedure [40]. All models incorporated carcass type within site as a random effect to account for repeated measures within sites. These same model sets were constructed again using ADD as the independent variable. If a top model incorporated carcass type, we substituted carcass type with carcass weight and added the new model to the model set to identify if carcass type effects were better explained by carcass size. Linear mixed effect modeling was conducted using function *lmer* (package lme4 v. 1.1; [44]) called by package lmerTest v. 2.0 [45]. Denominator degrees of freedom were estimated using Satterthwaite’s approximation, and significant effects were further explored using pairwise tests with P-values adjusted using a Bonferroni correction. Lastly, we qualitatively compared the proportion of carcasses that remained un-scavenged across experimental blocks to avoid an analysis with empty cells.

#### 1.3 Do patterns in local scavenger guild membership affect how carrion removal proceeds?

Carcass removal was characterized by two independent variables: proportion of each carcass that was consumed and carcass depletion time (i.e., time to last scavenger in hours). To determine if patterns in local scavenger guild membership affected carcass removal, we modeled independent variables separately against the dependent variables scavenger species richness and the scavenger responsible for opening each carcass (a categorical variable). Models incorporating the categorical variable were constructed as linear mixed effect models with season as a random effect. Models incorporating richness included linear models (*lm*), linear mixed effect models (*lmer*) using season as a random effect, and non-linear models (*nls*) constructed to test linear, logarithmic, exponential, and power relationships (S1 File). We used a model selection procedure as above for the sets incorporating richness to determine the best model for further interpretation.

## Results

We collected 36,466 images by monitoring carcasses with remote cameras. Data were excluded from 37 of 180 carcasses due to camera failures. These non-trials were unlikely to represent a systematic bias because they were evenly distributed across carcass types (raccoon = 14, opossum = 13, and rabbit = 10) and connectivity categories (low = 15, intermediate = 10, high = 12).

Of the 143 usable trials, 91.6% of carcasses (N = 131) were ≥ 50% consumed, 75.5% of carcasses (N = 108) were ≥ 90% consumed, 48.9% of carcasses (N = 70) were completely consumed, and 4.2% of carcasses (N = 6) remained intact for the month-long trial. Two of the six intact carcasses had brief scavenger activity that did not result in any apparent carcass depletion. Fourteen carcasses (9.8%) were opened and completely consumed by invertebrates, vertebrates were present and exhibited scavenging behavior at 125 carcasses (87.4%), and at four carcasses (2.8%) we did not observe evidence of scavenging by vertebrates or invertebrates. Vertebrates engaged in scavenging behavior for 493.99 hours across the 143 usable trials. Vertebrates spent more time (qualitatively) scavenging raccoon carcasses (273.55 hours) than rabbit carcasses (116.16 hours) and opossum carcasses (104.28 hours in total). Individual vertebrate species engaged in scavenging behavior for an average of 1.60 ± 0.17 hours per carcass (min = 0.08; median = 0.54; max = 31.77 hrs), and carcasses were active with scavengers for an average of 3.45 hours (± 0.41 SE; min = 0, median = 1.67; max = 34.48 hours) during trials.

Fifteen species were identified as vertebrate scavengers (Table 1). Guild composition was evenly divided by mammalian (N = 8) and avian species (N = 7). Mammals scavenged 106 carcasses, birds scavenged 88 carcasses, and mammals and birds scavenged 69 carcasses (Table 2). Opossums were the most frequent scavenger across carcass types, and the sum duration of opossum scavenging activity was 2.5 times longer than that of any other species (Table 1).

**Table 1:** List of vertebrate scavengers observed feeding on medium-sized carcasses in north-central Indiana, USA. Three carcass types (N = 60 of each) were deployed in one-month trials from September 2008—August 2009 and monitored with remote cameras. The number of carcasses each species scavenged is noted, and the duration in hours of scavenging by each species follows in parenthesis. Scavengers were scored as unknown in rare instances where the animal was obscured from view in the images.

		Carcass type			
Common name	Scientific name	Raccoon	Opossum	Rabbit	Total
Virginia opossum	*Didelphis virginiana*	36 (168.56)	24 (17.57)	31 (67.28)	91 (253.41)
Turkey vulture	*Cathartes aura*	21 (46.77)	20 (36.93)	10 (12.31)	51 (48.21)
Raccoon	*Procyon lotor*	11 (5.00)	9 (22.48)	24 (20.73)	44 (96.01)
Red-tailed hawk	*Buteo jamaicensis*	17 (38.20)	17 (18.40)	6 (5.45)	40 (62.05)
American crow	*Corvus brachyrhynchos*	13 (6.80)	8 (5.05)	9 (1.43)	30 (13.28)
Coyote	*Canis latrans*	7 (0.83)	4 (0.83)	5 (0.42)	16 (2.08)
Red fox	*Vulpes vulpes*	8 (2.03)	2 (0.25)	1 (0.08)	11 (2.37)
Dog (various breeds)	*Canis familiaris*	5 (0.58)	1 (2.77)	4 (3.67)	10 (7.02)
Unknown	.	2 (0.17)	0	4 (0.50)	6 (0.67)
Barred owl	*Strix varia*	1 (2.83)	0	1 (0.25)	2 (3.08)
House cat	*Felis catus*	0	0	2 (3.87)	2 (3.87)
American mink	*Neovison vison*	0	0	2 (0.17)	2 (0.17)
American robin	*Turdus migratorius*	1 (0.25)	0	0	1 (0.25)
Blue Jay	*Cyanocitta cristata*	1 (1.53)	0	0	1 (1.53)
Striped skunk	*Mephitis mephitis*	0	0	1 (0.17)	1 (0.17)
Yellow-bellied sapsucker	*Sphyrapicus varius*	0	0	1 (0.08)	1 (0.08)
Species per carcass type		12	8	14	-
Unique species per carcass type		2	0	4	-

**Table 2:** Frequency of vertebrate scavengers feeding on three different carcass types experimentally placed in study sites exhibiting three categories of patch connectivity. Frequency of mammalian, avian, or both types of vertebrate scavengers feeding on raccoon, opossum, and rabbit carcasses placed in habitat patches with low, intermediate, and high landscape connectivity in Indiana, USA.

	Connectivity	Carcass type			
		Raccoon	Opossum	Rabbit	Total
Mammalian	Low	10	7	12	29
	Intermediate	17	13	13	43
	High	12	9	13	34
	subtotal	39	29	38	106
Avian	Low	10	11	5	26
	Intermediate	11	10	6	27
	High	15	11	9	35
	subtotal	36	32	20	88
Both	Low	7	5	4	16
	Intermediate	11	9	6	26
	High	12	6	9	27
	subtotal	30	20	19	69
	Total available	46	47	50	143

### 2.1 Do connectivity and carcass type affect local scavenger guilds?

Richness of local vertebrate scavenger guilds (i.e., alpha diversity or the number of species/carcass) ranged from 0 to 6. Of those carcasses with vertebrate scavenging activity, mean richness (i.e., $\bar{\alpha }$ ± SE; [39]) was 2.46 ± 0.12 species. Three models of richness were competitive (i.e., ΔAIC < 4). The top model incorporated additive effects of carcass type (F_(2, 119)_ = 4.54, P = 0.013) and season (F_(3, 119)_ = 7.098, P < 0.001) on richness. Post-hoc tests indicated that raccoon carcasses harbored higher richness (2.72 ± 0.16 species) than opossum carcasses (2.12 ± 0.16; P = 0.011). Rabbit carcasses (2.44 ± 0.17) were no different from raccoon (P = 0.740) or opossum (P = 0.095) carcasses. Average richness in winter (3.00 ± 0.18) and spring (2.54 ± 0.20) was higher than in summer (1.76 ± 0.20; P < 0.001 and P = 0.039, respectively), but richness in winter, spring and summer was no different from fall (2.41 ± 0.17; P = 0.089, P = 0.963, and P = 0.095, respectively). The second best model (ΔAIC = 1.86) added a non-significant effect of connectivity (F_(2, 117)_ = 1.06, P = 0.351) to the parameters of the top model, offering no additional interpretative value [46], and the last competitive model (ΔAIC = 3.21) incorporated only the effects of season (F_(3, 121)_ = 7.46, P < 0.001). Therefore, richness was affected primarily by season and carcass type, but not by patch connectivity. We conducted an additional analysis to determine if carcass weight was driving the relationship between richness and carcass type. Analysis of covariance revealed that although carcass types differed in their average weight (raccoon [6.01 ± 0.18 kg] > opossum [2.70 ± 0.15] > rabbit [2.13 ± 0.04]; all P < 0.001), the effect of carcass type on richness was not diminished by partitioning-out variation in richness explained by carcass weight (carcass type: F_(2, 119)_ = 3.87, P = 0.023; carcass weight: F_(1, 119)_ = 0.58, P = 0.446; carcass type × carcass weight: F_(2, 119)_ = 0.01, P = 0.996).

**Fig 2:**
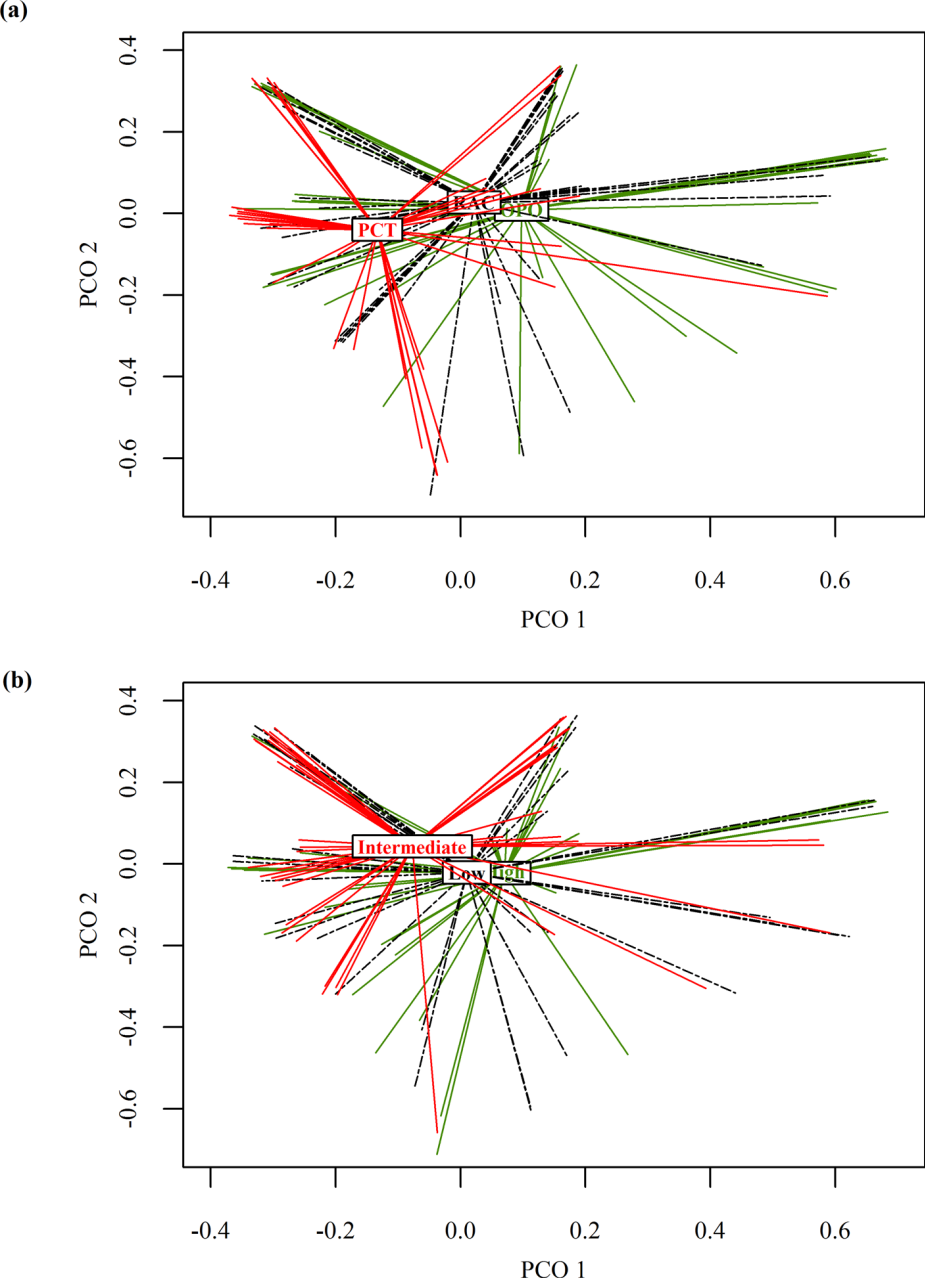
Centroid locations of local scavenger guild dissimilarity depicted by carcass type and by category of habitat patch connectivity. Dissimilarity measures were calculated for each carcass based on the presence/absence of species that engaged in scavenging behavior. Spider plots depict factor centroids for (a) carcass type (where RAC = raccoon, OPO = opossum, and PCT = rabbit) and (b) habitat patch connectivity resulting from principal coordinate analysis (PCO) using 1-Sorensen’s index as a measure of dissimilarity.

**Fig 3:**
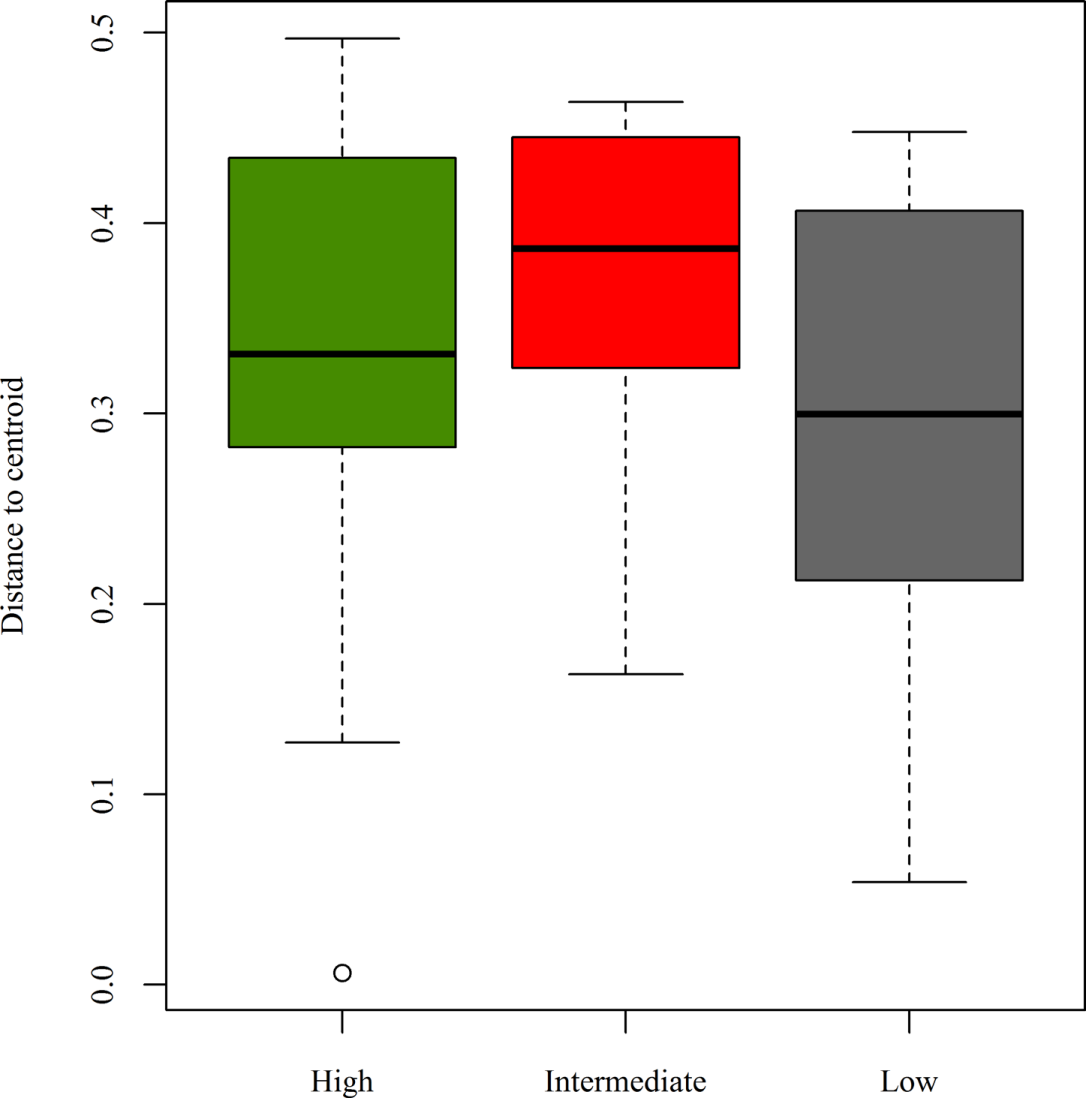
Dispersion of local scavenger guild dissimilarity by categories of habitat patch connectivity. Dispersion of dissimilarity measures calculated from local scavenger guilds that fed on carcasses deployed in patches with high, intermediate, and low levels of habitat connectivity in north-central Indiana, USA.

Average dissimilarity (i.e., beta diversity or ${\hat{\sigma }}^{2}$; Anderson et al. 2011) among local scavenger guilds was 0.609 (min = 0, max = 1, med = 0.6). Local scavenger guild centroids (*adonis*) were not significantly different across categories of patch connectivity (F_(2, 124)_ = 1.81, P = 0.053), but were affected by carcass type (F_(2, 124)_ = 4.71, P = 0.001) with no significant interaction (F_(4, 124)_ = 0.63, P = 0.729). Whereas most variation in local guild structure remained unexplained (residual R^2^ = 0.882), carcass type (R^2^ = 0.072) explained >2.5 times more variation in scavenger guild dissimilarity than patch connectivity (R^2^ = 0.027). Visualizing carcass type centroids (PCO), we identified rabbit carcasses separating from raccoon and opossum carcasses in multivariate space (Fig 2). Results were identical when using the Jaccard Index (data not shown). Analysis of group dispersions revealed that the near significance of the location effect for connectivity from *adonis* was likely due to a difference in dispersion, because connectivity (F_(2, 122)_ = 3.53, P = 0.032) but not carcass type (F_(2, 122)_ = 0.31, P = 0.073) drove patterns of multivariate variance in local guilds (*betadisper*; Anderson et al. 2006). Post-hoc analyses indicated that carcasses in sites with intermediate levels of patch connectivity had higher local guild variance than low connectivity patches (*TukeyHSD* post hoc tests; P = 0.025; Fig 3). Other pairwise comparisons were non-signficant (P > 0.05).

Dissimilarity in duration of scavenging activity by members of local guilds was influenced by season (P = 0.009), but not by habitat patch connectivity (P = 0.411) or carcass type (P = 0.510; *anova*.*manyglm* function in *mvabund*). Variation in dissimilarity among local guilds was driven by heightened durations of raccoon and red-tailed hawk scavenging in the winter relative to all other seasons (all P < 0.05), and increased scavenging activity by turkey vultures in spring and summer relative to winter (P < 0.001, and P = 0.040, respectively) and in spring relative to fall (P < 0.001).

The frequency with which invertebrate, avian, or mammalian species opened carcasses was unaffected by patch connectivity (χ^2^ = 6.410, df = 4, P = 0.755), but was affected by carcass type (χ^2^ = 13.901, df = 4, P = 0.008). Birds and invertebrates opened proportionally more opossum carcasses than did mammalian scavengers, whereas mammals opened proportionally more rabbit carcasses than birds and invertebrates (Table 3).

**Table 3:** Vertebrate scavengers that opened three carcass types. Frequency of scavengers that opened three different types of carcasses in study sites in Indiana, USA. The scavenger that opened a carcass was scored as unknown (likely a vertebrate) in rare instances where the camera images were inconclusive as to the species’ identity.

	Carcass type			
Opening scavenger	Raccoon	Opossum	Rabbit	Total
Mammalian				
Opossum	17	4	13	34
Raccoon	0	5	8	13
Coyote	1	0	1	2
Red fox	0	1	0	1
Avian				
Turkey vulture	10	12	5	27
Red-tailed hawk	8	8	4	20
Invertebrates	7	9	5	21
Unknown	1	1	3	5
Total	44	40	39	123

The probability that a carcass would be monopolized by invertebrates to the exclusion of vertebrate scavengers was not independent of carcass type and connectivity (likelihood ratio test: *χ*^2^ = 2.52, df = 4, P = 0.641; i.e., the interaction term was not significant). Single factor tests revealed that carcass type (*χ*^2^ = 7.00, df = 2, P = 0.030) but not connectivity (*χ*^2^ = 0.14, df = 2, P = 1.00) drove the lack of independence. Rabbit and opossum carcasses were 9 and 4 times more likely, respectively, to be monopolized by invertebrates than were raccoon carcasses.

### 2.2 Do connectivity and carcass type affect the timing and extent of carrion removal?

Time to the first scavenging event (in hours) was affected primarily by season. The best model (next best model ΔAIC = 138.9) for time to first scavenging was the full model containing all two-way and the three-way interactions, but season was the only significant parameter in the model (F_(3, 75.3)_ = 3.54, P = 0.019). Seasonal differences in time to first scavenging were driven by the difference between shorter summer latencies (99.3 ± 30.4 hours) and longer winter latencies (215.6 ± 25.2, df = 69.3, t = 3.00, P = 0.020). The ADD to first scavenger was similarly affected primarily by season. The full model of ADD to first scavenger again was most competitive (ΔAIC = 92.9) with season as the only significant parameter in the model (F_(3, 89.0)_ = 19.31, P < 0.001). Summer latency (50.76 ± 5.06 ADD) was significantly higher than that in fall (18.74 ± 3.98; df = 89, t = 4.97, P < 0.001), winter (1.09 ± 4.19; df = 89, t = 7.56, P < 0.001), and spring (24.23 ± 4.72; df = 89, t = 3.83, P = 0.001). Fall and spring ADD to first scavenger were also higher than winter (df = 89, t = 3.05, P = 0.018; df = 89, t = 3.67, P = 0.002, respectively).

Although first scavengers immediately opened carcasses in 68 of 143 trials, time to carcass being opened was still significantly longer than time to first scavenging on average (average difference = 21.16 hours; paired t-test df = 122, t = 2.845, P = 0.003). Mean time to carcass opening (in hours) was most affected by season. Season (F_(3,86.98)_ = 5.51, P = 0.001) was the only significant parameter in the saturated model (next best model: ΔAIC = 136.36). Summer latency to carcass opening (99.7 ± 30.2 hours) was significantly less than that of winter (261.8 ± 27.6; df = 86.9, t = 3.96, P = 0.001). Season was also the only significant parameter in the saturated model (next best model: ΔAIC = 96.4) of ADD to carcass opening (F_(3, 86.99)_ = 21.7, P < 0.001). ADD to carcass opening in fall (18.92 ± 3.72 ADD) was no different than that of spring (25.92 ± 4.41; df = 87, t = 1.21, P > 0.05), but all other pairwise comparisons revealed significant differences (summer: 51.39 ± 4.73, winter: 0.35 ± 4.32; all P < 0.05).

Random effects explained an insignificant amount of variation in mean time to the last scavenger, and all models were therefore evaluated without random effects using linear models. The best model of time to last scavenger (in hours) was the saturated model. The saturated model included significant effects of connectivity (F_(2,88)_ = 4.15, P = 0.019), carcass type (F_(2,88)_ = 6.95, P = 0.002), season (F_(3,88)_ = 10.40, P < 0.001), along with a significant three-way interaction (F_(12, 88)_ = 2.28, P = 0.014). Two-way interactions were not significant (P > 0.05). Other models in the top model set included a model with all single parameters (ΔAIC = 1.59), and a model containing all single parameters along with a non-significant two-way interaction between connectivity and season (ΔAIC = 3.72). We chose to interpret the top model due to the importance of the three-way interaction in explaining variation in time to last scavenger. Variation in time to last scavenger (in hours) was driven by 12 pairwise differences among carcass types, connectivity categories, and seasons (of 45 total). Seven of 12 significant comparisons involved the fastest (68.02 ± 64.84 hours) average depletion time which occurred in opossum carcasses within low connectivity patches during summer. Four of those seven comparisons involved pairings against longer depletion times for raccoon carcasses (estimate range 480.60–572.85 hours), and three of the raccoon pairings included data from winter (estimate range: 478.92–572.85 hours). The longest average time to depletion was for opossum carcasses in low connectivity sites during spring (574.11 ± 102.52 hours), highlighting the extensive variation in time to last scavenger that existed both within and across experimental blocks.

The best model for time to the last scavenger in ADD included carcass type (F_(2,100)_ = 12.79, P<0.001), connectivity (F_(2,100)_ = 8.25, P<0.001), and season (F_(3,100)_ = 40.30, P<0.001), along with each two-way interaction (all P<0.05; S2 Fig). The top model set also included the saturated model (ΔAIC = 0.624) with a non-significant three-way interaction, and a third model with the interaction parameters carcass type×season and connectivity×season (ΔAIC = 2.274). The top model set for time to last scavenger highlighted a disproportionate increase in latency to the last scavenger for raccoon carcasses as degree accumulation increased seasonally from winter (1.19 ± 11.91 ADD) to summer (158.59 ± 12.28) when compared with the same progression for opossum or rabbit carcasses (S2 Fig). Raccoon carcasses also drove patterns in time to last scavenger across categories of connectivity. Raccoon carcasses in intermediate connectivity sites (101.24 ± 9.57) had greater latency to last scavenger (in ADD) than all other carcass type × connectivity combinations, except for raccoon carcasses in low connectivity patches (71.17 ± 11.70; S2 Fig). Carcasses that remained intact (N = 6) were found only in winter (N = 4) and fall (N = 2). Opossum carcasses were left intact more often (N = 3) than rabbit (N = 2) or raccoon carcasses (N = 1), and sites with intermediate connectivity harbored more intact carcasses (N = 3) than isolated (N = 2) or connected (N = 1) sites.

### 2.3 Do patterns in local scavenger guild membership affect how carrion removal proceeds?

**Fig 4:**
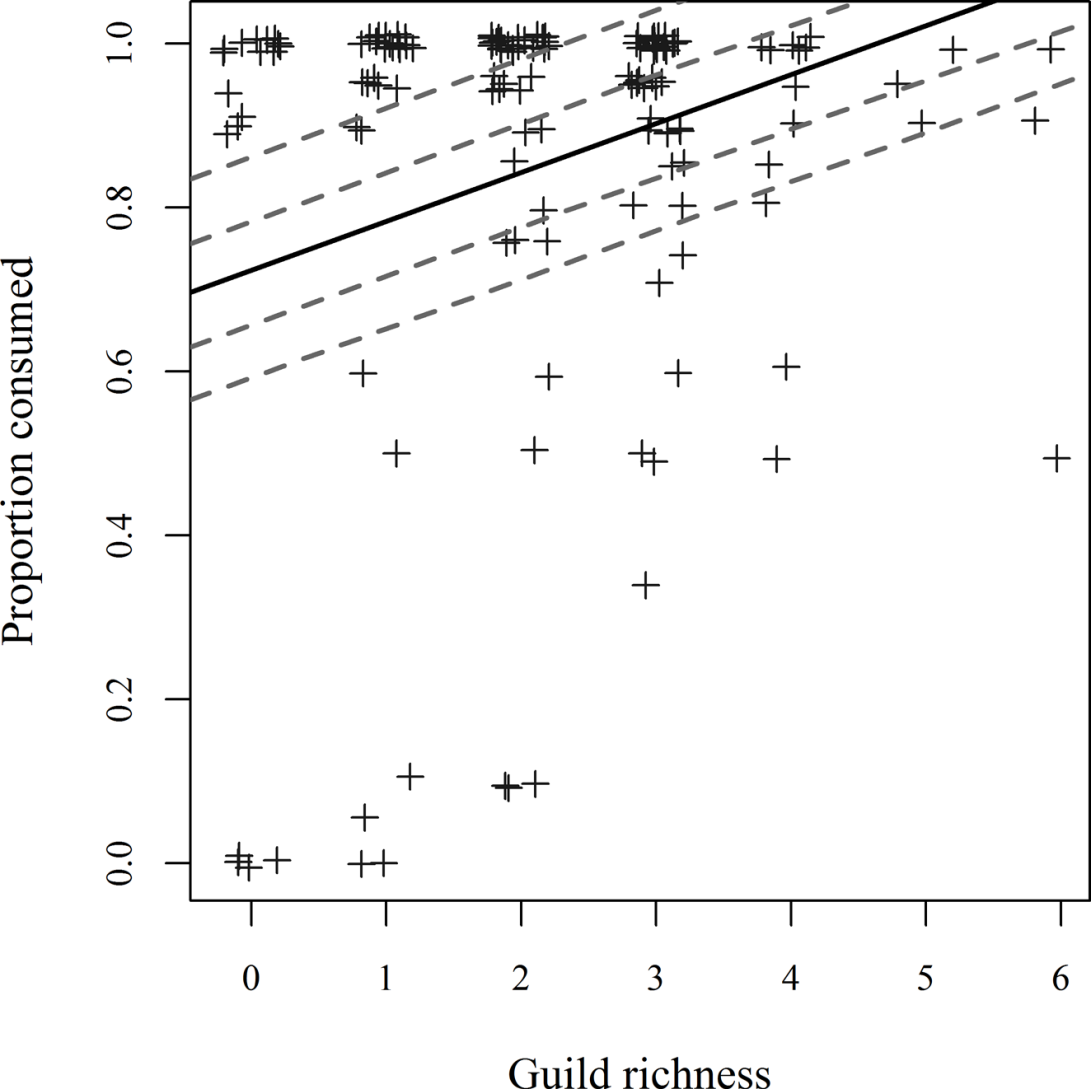
Linear relationship between carcass removal and richness of local vertebrate scavenger guild. Proportion of carcass consumed by the end of 1-month trials related to local vertebrate scavenger guild richness at each carcass. Hashed lines represent random intercepts for each season based on a linear mixed effect model. The solid line represents the fixed effect (estimate ± 1SE = 0.0597 ± 0.0170).

In all cases a linear mixed effect model incorporating season as a random effect was the most parsimonious model in the top model set. Average time (in hours) to carcass depletion was not affected by the species responsible for opening the carcass (F_(8, 116.03)_ = 0.888, P = 0.529) or by richness of the local scavenger guild that assembled to feed on the carcass (F_(1, 124.89)_ = 2.884, P = 0.092). The proportions of carcass consumed during trials did not differ whether the carcass was opened by invertebrate, mammalian, or avian species if analyses were restricted to warmer months when all three categories of animals were competitive for carcass resources (species combined into categories to remove empty cells; F_(2, 62.942)_ = 1.089, P = 0.343). However, every one-scavenger increase in richness at a carcass resulted in a 6% increase in carcass consumption (F_(1, 140.94)_ = 12.354, P<0.001; Fig 4).

## Discussion

Utilization of carrion within an agricultural ecosystem was strongly influenced by the type of carcass deployed (raccoon, opossum, or rabbit) and was much less affected by the degree of landscape connectivity where the carcass was deployed (low, intermediate, and highly connected patches). Although richness of local scavenger guilds [6] in our study (≈2.5 species on average) was comparable to that of other studies (≈3.1–3.5 [47] and ≈2.0–4.8 species [8]), average richness was 24% lower for opossum carcasses than for raccoon carcasses but was not different across categories of patch connectivity. Further, whether a carcass was opened (i.e., skin perforated) by a mammal, bird, or by invertebrates was also dependent only on carcass type and not patch connectivity, with mammals opening more rabbit carcasses and birds and invertebrates opening more opossum carcasses. We found little support for our hypothesis that landscape arrangement would affect membership of local scavenger guilds more than the type of carcass. In contrast, the density of feeding stations on the landscape, among other variables, was shown to influence relative abundances within local guilds of four species of obligate-scavenging vultures in Europe [48].

The idea that the species identified during scavenging trials should be considered representative of the larger vertebrate community aligns well with evidence suggesting that scavenging behavior is widespread among vertebrates [3]. As such, carcasses might be expected to assemble similar local guilds across trials within a landscape [23]. However, local guilds in our study exhibited dissimilarity in species occurrence that was driven primarily by carcass type after accounting for seasonal variation, with rabbit carcasses separating from raccoon and opossum carcasses in multivariate space. Raccoons and opossums probably contributed to this separation by reducing their scavenging proportionally at conspecific carcasses (Table 1). Disease transmission likely plays an evolutionary role in a species’ innate aversion to particular carcasses [4,49], and could be especially relevant here. The relative role of innate and learned responses as they apply to the foraging decisions of individual scavengers is an area worthy of future investigation.

A potential confound in our study design was a known relationship between carcass type and carcass size (average weight of raccoon > opossum > rabbit carcasses). Carcass size has been shown to effect local scavenger guild structure and function [8,50,51]. Although not the case for our study, one route by which carcass size can affect scavenging is when carrion becomes proportionally larger than the scavenger (see [52]). Many potential vertebrate scavengers lack the size, power, or ability to access resources from intact large carcasses, but are able to scavenge effectively once a carcass has been opened to reveal more manageable pieces [53]. Despite the fact that carcass type was confounded with carcass weight in our study, when addressed statistically carcass weight was never as informative as carcass type in describing variation in measures of the scavenger community or of carcass removal. The idea that carcass type might matter to scavengers has conservation implications where species of obligate-scavenging vultures are provisioned with livestock and wild prey carrion (e.g., in Europe). For example, Moreno-Opo et al. [54] concluded that providing ovine or caprine carcasses, rather than bovine or wild carrion, in pieces resulted in greater feeding opportunities for their species of conservation concern.

Once scavengers committed to feed on a carcass, their duration of scavenging activity was not affected by the type of carcass or by patch connectivity. However, scavengers did exhibit seasonal variability in their activity at carcasses with raccoons and red-tailed hawks scavenging for greater durations in winter relative to other seasons. Turkey vultures, the only obligate scavenger and a seasonal resident in our study area [55], not surprisingly scavenged for greater durations during spring relative to fall and winter and in summer relative to winter. Past research has identified the importance of carrion in providing critical resources during lean seasons [51,56,57]. Our data support this idea and provides complementary evidence that the absence of a migratory obligate scavenger during colder months may release resources to resident animals.

Obligate scavenging vultures are well adapted to win scramble competitions for carrion resources [58,59], and the turkey vulture in our study was an influential member of local scavenger guilds. However, other forms of competition may also be important in shaping the structure of local scavenger guilds. Dominance hierarchies among old world vultures are well characterized [60] as is the fact that carcasses can be the focus of interspecific contest competitions ([3,5,47,51]). Competitive interactions probably shape local scavenger guild structure [61], but carrion removal seems to be preserved as an ecosystem service by different local scavenger guilds at different times of the year or across habitats [25].

Separate from the larger effect of carcass type on local guild dissimilarity, we also found a weaker effect of connectivity on local scavenger guild assemblages, but the effect was one of dispersion rather than location. Trials in sites with intermediate levels of patch connectivity exhibited more variability in guild dissimilarity measures than did isolated sites, meaning that carcasses at intermediate sites in some trials attracted local scavenger guilds that were very similar to the average local guild and in other trials were distinct from the average local guild. We do not have an explanation for why this variation might have occurred, especially given the fact that vertebrate scavenger species richness was the same at sites with intermediate connectivity (n = 11) as it was at low (n = 12) and high connectivity sites (n = 13).

A focus on the temporal aspects of carrion removal yielded a nuanced picture of how carrion removal proceeds. Contrary to our predictions, the type of carcass deployed and the level of patch connectivity at a site did not affect the amount of time that elapsed until scavengers found a carcass and the time it took animals to open a carcass. Time to first scavenger and time to carcass opening exhibited only seasonal variation with both processes occurring predictably more slowly (in hours and additive degree days) in colder seasons than in warmer seasons.

The use of additive degree days (ADD) as a temporal measure of scavenging processes has intuitive appeal, because many scavenging related phenomena are known to be temperature-dependent [3]. For example, volatile compounds that serve to attract invertebrate and vertebrate scavengers are produced by microbial action, and microbial action in carcasses is mediated primarily by temperature [62]. Similarly, feeding by invertebrate larvae strongly influences the fate of carrion, and rates of larval development are also temperature dependant [63].

Despite the intuitive appeal of ADD as a metric, we developed no clearer picture of the temporal aspects of carrion removal when time was modeled in ADD rather than in hours. For example, we considered our measure of carcass depletion (time to last scavenger) to be important from the perspective of potential disease transmission if some carcasses persisted on the landscape for longer durations, or if carcasses in some landscape contexts remain longer than others. However, time to carcass depletion exhibited substantial variation within carcass types, connectivity categories, and seasons when measured in both hours (significant three-way interaction) and ADD (all two-way interactions significant; see Results). A potential explanation for this variation can be found in the forensics literature, which highlights the importance of micro-site parameters in determining rates of decomposition. For example, relative differences among carcasses in exposure to sun or to moisture can speed or slow decomposition substantially [12]. It seems likely that micro-site variations in ADD that went unmeasured in our study contributed to variation around carcass depletion.

Seasonal effects, on the other hand, explained variation across several measures of local scavenger guild diversity and across measures of carcass depletion. Carcasses only remained un-depleted by scavengers during trials in and fall and winter, when average temperatures were low (mean ± SE; -4.4 ± 0.6°C, 0 ADD) and invertebrates were inactive. Other investigations have also found that carcass removal is less efficient when invertebrates are absent or excluded [64,65]. Although relatively infrequent overall (4.2% of carcasses), the rate of intact carcasses at the end of trials in our study matched the rate from shorter deployments of mouse (*Mus musculus*) carcasses in the same study area (6.8%; $\chi_{1}^{2}$ = 1.18, P = 0.278; [10]). However, our rate of intact carcasses differed from previous work when smaller carcasses were deployed into experimentally disrupted scavenger communities (10.5%; $\chi_{1}^{2}$ = 4.83, P = 0.028; [10]). Adding to the idea that intact and species-rich communities are more efficient at carrion removal [61,66], our results indicate that increasing richness of local vertebrate scavenger guilds contributes moderately to the reliable delivery of carcass removal as an ecosystem service.

Disassembling the effects of species diversity from functional diversity can be an effective way to learn about complex ecological phenomena [67]. Scavenging is one such phenomenon, and exists as a dynamic feeding relationship through which the ecosystem service of carrion removal is achieved. Several important points emerged from our study that could be useful in future research on scavenging ecology. First, the nature of the carcass itself seems to be an important factor contributing to a potential scavenger’s resource utilization choice. This implies that studies of scavenging ecology must account for the potential that scavenger-specific differences in carrion utilization exist among carcass types. Second, seasonal changes in carrion utilization, presumably driven by temperature, not only affect the probability of carrion removal over time but also the diversity of species that may utilize the resource. Lastly, carcass removal was tied to both the temperature-dependent activity of invertebrates and to the richness of local vertebrate scavenger guilds. Thus, reliable delivery of carrion removal as an ecosystem service may depend on robust, synergistic interactions between vertebrate and invertebrate communities.

## Supporting Information

S1 Fig Study sites binned by index of proximity. (PDF)

S2 Fig Relation of additive degree days (ADD) to carcass type and patch connectivity. (a) Additive degree days to last scavenger increased at different rates for different carcass types as seasonal temperatures moved from cold to warmer. (b) Carcass types also differed by season in their average time (in ADD) to depletion.
(PDF)

S1 Dataset Summarized scavenging data from Indiana, USA. (XLSX)

S1 File Model structures to address Objective 3. (PDF)
