# Supplementary figures and images for: Carcass Type Affects Local Scavenger Guilds More than Habitat Connectivity
Source: PLoS One. 2016 Feb 17;11(2):e0147798. doi: 10.1371/journal.pone.0147798 (PMC4757541; doi:10.1371/journal.pone.0147798)

S1 Figure.

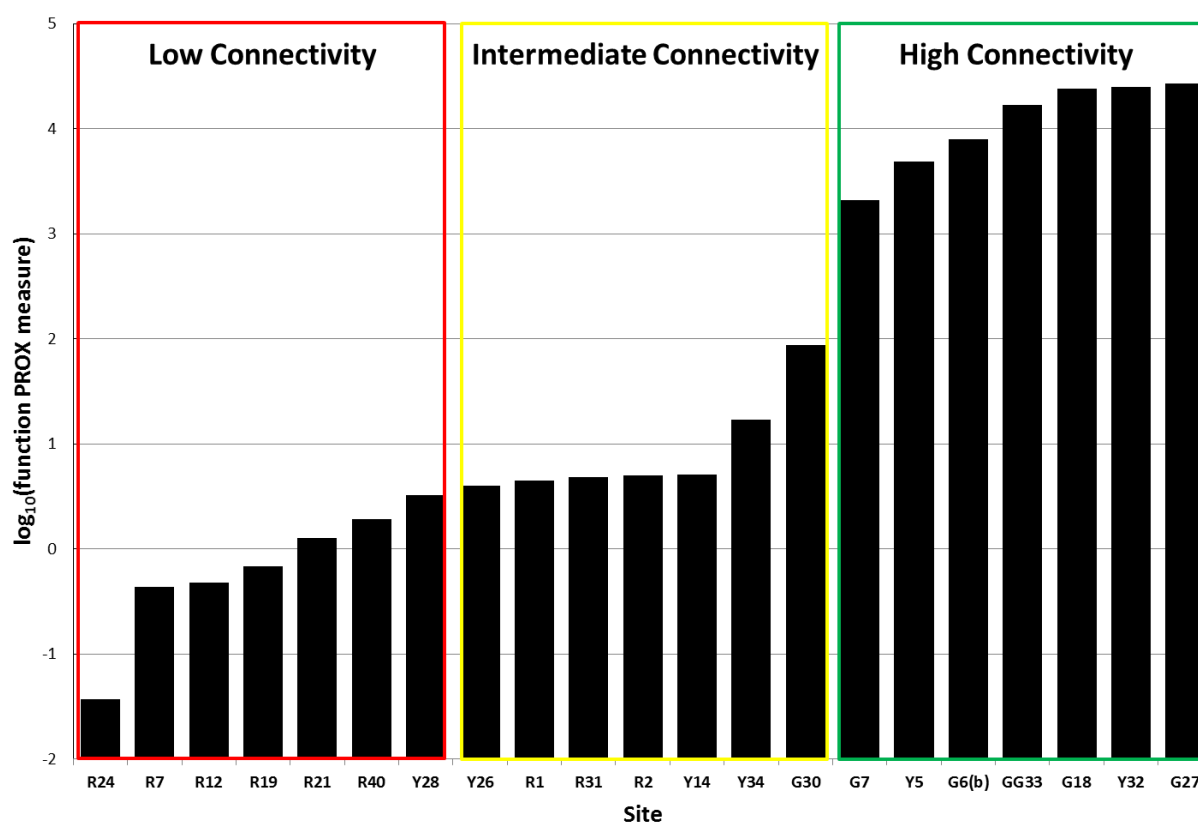

Supplement: S1 Fig — (PDF) [file pone.0147798.s001.pdf]

S2 Figure.

a)

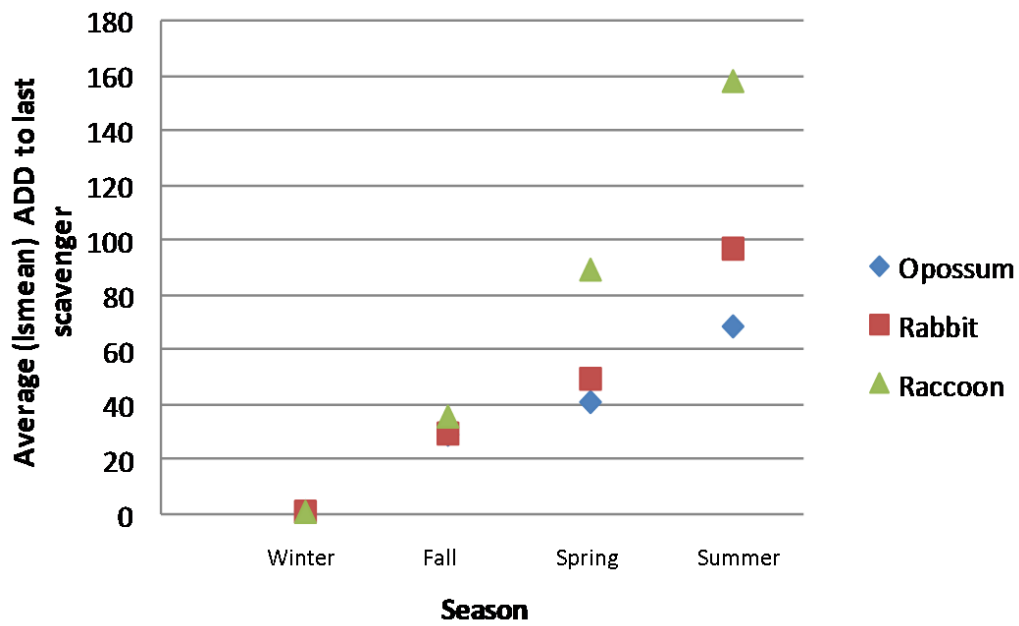

b)

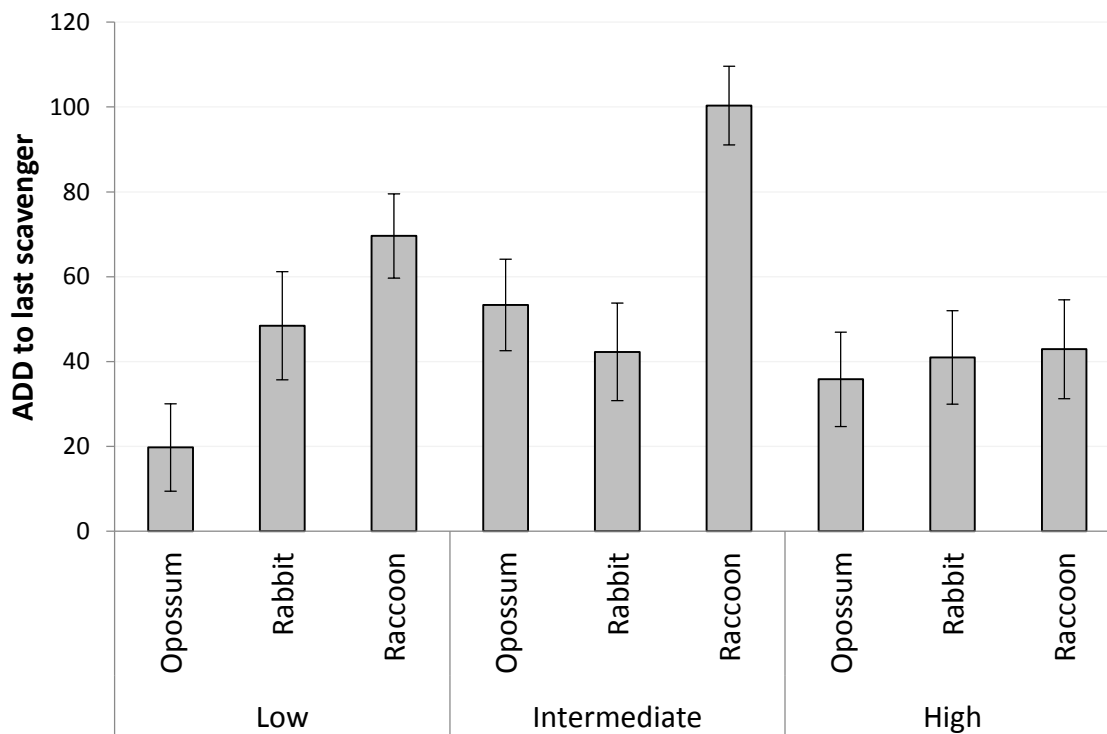

Supplement: S2 Fig — (a) Additive degree days to last scavenger increased at different rates for different carcass types as seasonal temperatures moved from cold to warmer. (b) Carcass types also differed by season in their average time (in ADD) to depletion. (PDF) [file pone.0147798.s002.pdf]
