# Supplementary material for: Carcass Type Affects Local Scavenger Guilds More than Habitat Connectivity
Source: PLoS One. 2016 Feb 17;11(2):e0147798. doi: 10.1371/journal.pone.0147798 (PMC4757541; doi:10.1371/journal.pone.0147798)
Supplement: S1 File — (PDF) [file pone.0147798.s004.pdf]

S1 Supporting Information.

Model structures to address Objective 3: Do patterns in local scavenger guild membership affect how carrion removal proceeds?

Question 1) Does time to last scavenger (our measure of carcass depletion) depend on the species that opened the carcass? Results were not different if ADD was used instead of time in hours.

Model type	Parameters	Random effects
linear mixed effect model	Time to last scavenger ~ species that opened the carcass	Season

Results:

```
               Df Sum Sq Mean Sq F value  Denom Pr(>F)
carcass.opened  8 205883   25735  0.8878 116.03 0.5291
```

Question 2) Does time to last scavenger depend on species richness of local scavenger guilds? Results were no different if ADD was used instead of time in hours.

Model type	Parameters	Random effects	df	AIC
Linear mixed effect model	Time to last scavenger ~ richness	Season (intercepts)	4	1674.066
Linear mixed effect model	Time to last scavenger ~ richness	Season (intercepts) + Richness within Season (slopes)	5	1676.066
Logarithmic model	Time to last scavenger ~ a + b*(log(richness+0.01)) where a and b were found by iteration using *nls*	-	3	1690.955
Linear model	Time to last scavenger ~ richness	-	3	1692.843
Exponential model	Time to last scavenger ~ exp^(a + b(richness)) where a and b were found by iteration using *nls*	-	3	1694.029
Power model	Time to last scavenger ~ a+ richness^(b) where b was found by iteration using *nls*	-	3	1699.045

Question 3) Does the proportion of each carcass consumed at the end of trials depend on the organism that opened the carcass? To eliminate empty cells we grouped individual species into the categories "Avian", "Mammalian", and "Invertebrates", and data were limited to only trials where invertebrates were active (ADD at last scavenging > 20).

Model type	Parameters	Random effects
Linear mixed effect model	Proportion consumed ~ organism that opened the carcass	Season

Results:

```
             Df   Sum Sq  Mean Sq F value  Denom Pr(>F)
car.opened  2 0.025647 0.012824  1.0895 62.942 0.3426
```

Question 4) Does the proportion of each carcass consumed at the end of trials depend on the richness of the local scavenger guild that assembled to feed on it?

Model type	Parameters	Random effects	df	AIC
Linear mixed effect model	Proportion consumed ~ richness	Season (intercepts) + Richness within Season (slopes)	5	30.19
Linear mixed effect model	Proportion consumed ~ richness	Season (intercepts)	4	31.94
Power model	Proportion consumed ~ a+ richness^(b) where b was found by iteration using *nls*	-	3	33.72
Logarithmic model	Proportion consumed ~ a + b*(log(richness+0.01)) where a and b were found by iteration using *nls*	-	3	33.75
Linear model	Proportion consumed ~ richness	-	3	34.05
Exponential model	Proportion consumed ~ exp^(a + b(richness)) where a and b were found by iteration using *nls*	-	3	34.18

Results for Proportion consumed ~ richness + (1|season):

```
Analysis of Variance Table of type 3 with  Satterthwaite  approximation for
degrees of freedom
         Df  Sum Sq Mean Sq F value  Denom    Pr(>F)
richness  1 0.75781 0.75781  12.354 140.94 0.0005921 ***
```
